# Factors Associated with Mutations: Their Matching Rates to Cardiovascular and Neurological Diseases

**DOI:** 10.3390/ijms22105057

**Published:** 2021-05-11

**Authors:** Hannah B. Lucas, Ian McKnight, Regan Raines, Abdullah Hijazi, Christoph Hart, Chan Lee, Do-Gyoon Kim, Wei Li, Peter H. U. Lee, Joon W. Shim

**Affiliations:** 1 Department of Biomedical Engineering, College of Engineering and Computer Sciences, Marshall University, Huntington, WV 25755, USA; painter56@marshall.edu (H.B.L.); mcknight31@marshall.edu (I.M.); raines83@marshall.edu (R.R.); hijazia@marshall.edu (A.H.); hart121@marshall.edu (C.H.); 2 Indiana University Health Arnett Hospital, Lafayette, IN 47905, USA; clee9@iuhealth.org; 3 Division of Orthodontics, College of Dentistry, The Ohio State University, Columbus, OH 43210, USA; kim.2508@osu.edu; 4 Department of Biomedical Sciences, Joan C. Edwards School of Medicine of Marshall University, Huntington, WV 25755, USA; liwe@marshall.edu; 5 Department of Cardiothoracic Surgery, Southcoast Health, Fall River, MA 02720, USA; peter_lee@brown.edu; 6 Department of Pathology and Laboratory Medicine, Warren Alpert Medical School, Brown University, Providence, RI 02912, USA

**Keywords:** hypertension, congenital heart disease, thoracic aortic aneurysm, telomere, adenine thymine content, single nucleotide polymorphism (SNP), Parkinson’s disease

## Abstract

Monogenic hypertension is rare and caused by genetic mutations, but whether factors associated with mutations are disease-specific remains uncertain. Given two factors associated with high mutation rates, we tested how many previously known genes match with (i) proximity to telomeres or (ii) high adenine and thymine content in cardiovascular diseases (CVDs) related to vascular stiffening. We extracted genomic information using a genome data viewer. In human chromosomes, 64 of 79 genetic loci involving >25 rare mutations and single nucleotide polymorphisms satisfied (i) or (ii), resulting in an 81% matching rate. However, this high matching rate was no longer observed as we checked the two factors in genes associated with essential hypertension (EH), thoracic aortic aneurysm (TAA), and congenital heart disease (CHD), resulting in matching rates of 53%, 70%, and 75%, respectively. A matching of telomere proximity or high adenine and thymine content projects the list of loci involving rare mutations of monogenic hypertension better than those of other CVDs, likely due to adoption of rigorous criteria for true-positive signals. Our data suggest that the factor–disease matching rate is an accurate tool that can explain deleterious mutations of monogenic hypertension at a >80% match—unlike the relatively lower matching rates found in human genes of EH, TAA, CHD, and familial Parkinson’s disease.

## 1. Introduction

Hypertension remains the single biggest risk factor contributing to the global burden of disease and mortality [1]. Despite the prevalence of individuals with elevated blood pressure, the role of genetics in hypertension is poorly understood. Butler cited that 30%–50% of the variance of blood pressure readings is attributable to genetic heritability and about 50% is attributable to environmental factors [2]. With the advent of new molecular techniques, genetic mutations linked to monogenic hypertension (MH) have been found, suggesting that understanding the pathologies of these monogenic disorders provides insight into the causes of the more prevalent essential hypertension (EH) and new avenues to unravel the complexities of blood pressure regulation [3]. We have recently demonstrated that mutations causative of the congenital disorder can be projected by a stochastic approach centered on chromosomal characteristics of human genomes [4]. Germline mutations, which are created by homologous recombination, are estimated to occur in humans with an average probability of 1.28 × 10^−8^ per site per generation, with ∼93% of these being point mutations [5,6]. Germline point mutations can result in the creation of single nucleotide polymorphisms (SNPs) in a population [7]. Mutations have been proposed as natural variations that contribute to both intraspecies diversity (human genome) and interspecies divergence [8,9,10,11]. Several factors associated with these mutations have been implicated, including whether they are advantageous, neutral, or deleterious in human genomes and/or between species [8,9,12]. Excluding the impact of the father’s age at conception [13], there is an estimated 63–65 mutations per generation in humans, with 1–2 of these being considered deleterious.

Genes that satisfy one or more of these criteria are more likely to undergo mutations in the next generation. However, whether these mutations are advantageous, neutral, and/or deleterious is not determinable until the phenotype has been confirmed. Genes can be categorized as being causative of the disease or associated with the disease. Genes are considered causative if they can be directly linked to the expected phenotype, often determined in model systems with a phenotype that is comparable to that seen in humans. On the other hand, associated genes are those in which the cause and effect of the gene and its phenotype cannot be explained by genetic factors alone. Genes associated with essential hypertension (EH) and Alzheimer’s disease [4] are valid examples of how genetic and environmental factors may both contribute to the disease phenotype. In this study, we also use the term ‘genetic susceptibility’ when we refer to a candidate gene susceptible to the disease with an additional contributor [14]. 

Arterial stiffening, an independent predictor of cardiovascular morbidity and mortality, plays an important role in cardiovascular diseases (CVDs) [15,16], including hypertension [17,18], congenital heart disease (CHD) [19], and thoracic aortic aneurysms (TAA) [20,21]. Given the multigenic causes of these conditions, elucidation of the genetic characteristics or factors impacting high mutation rates might reduce the burden of high mortalities [22] in these CVDs. Furthermore, understanding the inherited genomic characteristics associated with arterial stiffening might lead to improved diagnosis and treatment of hypertension, CHD, and TAA. This idea, along with increasing evidence from human genome sequencing, suggests that select CVDs such as hypertension, CHD, and TAA most likely result from the interaction of various genetic and environmental cues [23,24,25,26,27,28,29]. 

Several factors have been reported to be associated with high mutation rates, including recombination rate, proximity to a telomere (F(i)) [30], and high A+T content (F(ii)) [9,12,31]. Among these factors, we have previously shown that F(i) [8] and F(ii) can explain some of the genetic mutations linked to monogenic and/or degenerative disorders [4]. However, whether these factors can provide a quantitative clue by percent on the genetic and/or environmental contributions to CVDs remains unclear. 

Telomeres—which are essentially characterized by a unique chromatin environment [30]—are composed of 3–20 kb of A+T rich repeats flanked by subtelomeric regions extending up to 300 kb into the chromosome [32]. Furthermore, tandem repeats of short GT-rich sequences are characteristic of almost all eukaryotic telomeres [33]. Specifically, human telomeres range in size from 2 to 50 kb and consist of 300–8000 precise repeats of the sequence CCCTAA/TTAGGG. A common feature of all telomeres is the orientation of the G-rich strand. In all cases, this strand comprises the 3′-end of the chromosome, while its terminal part is single-stranded, generating a G-tail [34]. 

Here, we investigate the two aforementioned chromosomal factors to determine whether they are associated with high mutation rates in human genes related to CVDs. By analyzing the sets of genes published previously for hypertension, CHD, and TAA, we aim to determine how many genes satisfy the two factors F(i) and F(ii) [4,12]. To do so, we compare the matching rate of these two criteria to the disease, focusing on the gene transcripts and/or genes near the loci involving coding and non-coding regions obtained from four independent prior studies. We provide the factor–disease matching rate of EH, which a majority of individuals with hypertension have. Then, we assess how many genes near the loci involving >25 rare mutations and 53 SNPs contributing to the genetic architecture of hypertension meet F(i) or F(ii), leading to an alternative conclusion. Subsequently, we demonstrate whether mutations causing CHD would satisfy either F(i) or F(ii). To this end, we show how genes associated with TAA reveal the factor–disease matching rate and provide a comprehensive summary of genomic features associated with mutations in CVDs as compared to those of neurological disorders. 

## 2. Results

### 2.1. Forty-One Genes Associated with Idiopathic or Essential Hypertension (EH)

**Figure 1:**
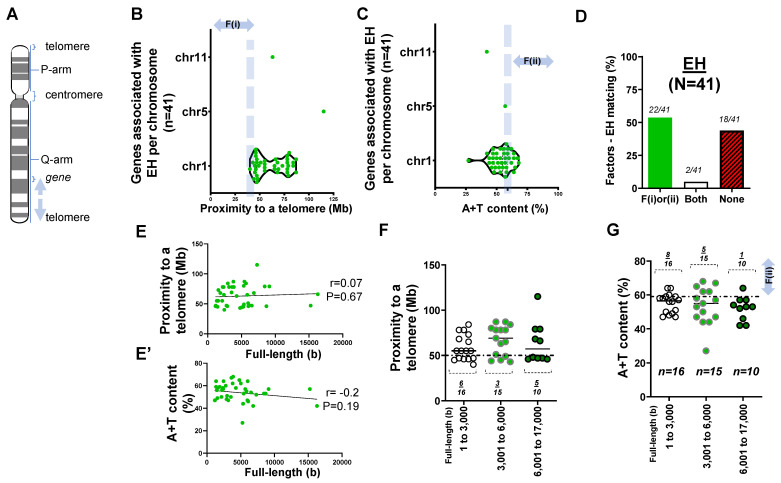
Genes associated with essential hypertension (EH) from microsatellite analysis. (**A**) A cartoon illustrating a distance to a telomere (arrows) from a gene in a human chromosome [12]. (**B**) Box and violin plots showing the distribution of 41 genes associated with EH with respect to proximity to telomeres over chr 1 to 11. F(i) indicates the first factor, namely proximity to the telomere. (**C**) Box and violin plots summarizing the full distribution of genes associated with EH with respect to A+T content over chr 1, 5, and 11. F(ii) indicates the second factor, namely high A+T content at >59%. (**D**) Bar graph demonstrating factors–EH matching rate. (**E**) Scatter plot exhibiting the Pearson correlation between the full length (base or b) of genes associated with EH and their proximity to telomeres. (**E’**) Scatter plot showing the Pearson correlation between the full length of genes associated with EH and A+T content. (**F**) Scatter plot showing proximity to telomeres of genes associated with EH with respect to three subgroups of the gene full-length size. A horizontal dotted line indicates 50 Mb. (**G**) Scatter plot summarizing A+T content of genes associated with EH with respect to the gene size in base or b. A horizontal dotted line indicates 59%. The statistical difference after one-way ANOVA among three groups of 1 to 3000, 3001 to 6000, and 6001 to 17,000 at *p* > 0.05 (**F**,**G**).

In assessing the list of the proposed genes of EH in Table S1, we surveyed the distance between each gene locus and its telomere using the F(i). As we identified 41 gene loci listed in Table S1, the candidate genes of EH are mostly skewed towards chromosome one (Figure 1A,B). 

To further assess this notion, we obtained the A+T content of genes associated with EH. Genes associated with EH rarely met high A+T content at >59% (Figure 1C). As we identified the relationship between the two aforementioned factors and the disease, ~53% of genes satisfied the proximity to telomeres or high A+T content (*n* = 22/41). Intriguingly, almost half of the genes (18 of 41) associated with EH met neither F(i) nor F(ii). Furthermore, only 2 of 41 genes met both F(i) and F(ii) (Figure 1D).

During analysis of the DNA composition of each gene, we asked if the full-length size of a gene, if it was larger than 6000 bp, was related to A+T content. The Pearson coefficient (r = 0.07) indicated that there was no significant correlation (*p* = 0.67) between the full-length sizes of the genes and proximity to telomeres in EH. We found no significant correlation between the full-length size and A+T content (r = −0.2; *p* = 0.19) (Figure 1E,E’).

We then evaluated factors (i) and (ii) with respect to the base pair of each gene associated with EH. We grouped these forty-one genes into three categories: 1–3000 bp (*n* = 16), 3001–6000 bp (*n* = 15), and 6001–17,000 bp (*n* = 10) (Table S1). Statistical analysis suggested that there was no significant difference in proximity to telomeres with respect to the gene full-length size (*p* > 0.3). Furthermore, pairwise comparisons using Tukey’s post hoc test after one-way ANOVA suggested that there was no significant difference (*p* > 0.5) in A+T content with respect to the full-length size (bp) between the groups (Figure 1F,G). 

Overall, the mismatch of two factors and the disease (EH) as well as the correlation between the full-length size of the genes and A+T content was either unexpectedly low (~53%) or statistically insignificant. 

### 2.2. Rare Mutations and SNPs Contributing to the Genetic Architecture of Hypertension

**Figure 2:**
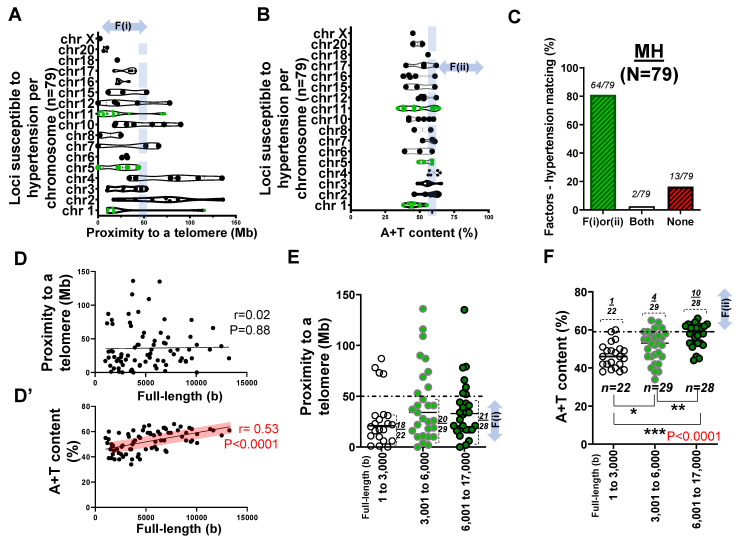
Genes (*n* = 79) near the loci susceptible to MH from exome sequencing and GWAS/SNPs analysis. (**A**) Box and violin plots showing the distribution of 79 genes near the loci proposed as candidates for hypertension [35] with respect to proximity to telomeres over chr 1 to X. The candidate genes are widely distributed over almost all chromosomes. F(i) indicates the proximity to the telomere. (**B**) Box and violin plots summarizing the full distribution of genes susceptible to hypertension with respect to A+T content. F(ii) indicates high A+T content at >59%. (**C**) Bar graph demonstrating factors–hypertension matching rate. (**D**) Scatter plot exhibiting the Pearson correlation between the full length of genes susceptible to hypertension and their proximity to telomeres. (**D’**) Scatter plot showing the Pearson correlation between the full length of genes susceptible to hypertension and A+T content. Pearson correlation at r = 0.53 with the level of statistical significance at *p* < 0.0001. (**E**) Scatter plot showing proximity to telomeres of 79 genes susceptible to hypertension with respect to three subgroups of the gene full-length size (unit: base or b). A horizontal dotted line indicates 50 Mb. (**F**) Scatter plot summarizing A+T content of genes susceptible to hypertension with respect to the gene size in base or b. A horizontal dotted line indicates 59%. The statistical difference after one-way ANOVA among three groups of 1 to 3000, 3001 to 6000, and 6001 to 17,000 at *p* < 0.05. * *p* < 0.05, ** *p* < 0.01, *** *p* < 0.005.

With a myriad of intriguing mismatches between the two considered factors and EH obtained through microsatellite markers, we asked what differed in the EH study of Figure 1 as compared to the previous investigations on CNS disorders [4]. Using the refined literature search mentioned in the methods section, we investigated the position of 79 genes near the loci proposed for hypertension (Table S2). In assessing the list of genes susceptible to hypertension in Table S2, we found that the list of >25 rare mutations (monogenic) and 53 SNPs were distributed over almost all chromosomes (Figure 2A). Our observation of the human chromosomes confirmed that susceptible genes located in chr 18, 19, and 20 were more prone to mutations because they more likely satisfied F(i) than genes in other chromosomes. 

To further test our hypothesis regarding F(ii), we obtained the A+T content of genes near the loci proposed for hypertension. When we examined 79 genes susceptible to MH and contributing to the genetic architecture of hypertension focusing on the factor–disease matching rate, >81% exclusively satisfied either the F(i) or F(ii) (*n* = 64/79) condition. Unlike the previous study (Figure 1C), a quarter of these genes displayed high A+T content at higher than 59% (Figure 2B). Sixteen percent of genes (13 of 79) associated with hypertension met neither F(i) nor F(ii). Furthermore, 2 of 79 genes met both F(i) and F(ii) (Figure 2C). This lends stronger support to the idea that if there is a match between the factor and the disease, telomere proximity or A+T content will suffice to explain deleterious mutations and SNPs in both coding region and non-coding RNA (>81% match) (Figure 2C). 

The Pearson coefficient of r = 0.02 suggested that there was no significant correlation (*p* = 0.88) between the full-length size and the F(i) in hypertension (Figure 2D). However, the Pearson coefficient between the full-length size and F(ii) (r = 0.53) suggested that there was a significant correlation (*p* < 0.0001) (Figure 2D’; highlighted in color).

We then organized genes according to two factors with respect to the base pair length. Consistent with the previous analysis [4], we grouped genes into three categories: 1–3000 bp (*n* = 22), 3001–6000 bp (*n* = 29), and 6001– 17,000 bp (*n* = 28). We found that there was no significant correlation between the F(i) and the full-length size of the gene (*p* > 0.05) (Figure 2E). However, the pairwise comparisons suggested that there was a significant correlation (*p* < 0.0001) between F(ii) and the full-length size (bp) when comparing three groups sorted by the nucleotide size (Figure 2F).

### 2.3. Mapping of 57 Genes Causing CHD in Human Chromosomes

In our search of congenital cardiovascular defects, we found a recent report linking cilia to heart defects, claiming that the likelihood of congenital heart disease (CHD) is up to 10-fold higher in human fetuses, affecting nearly 1% of live births [36,37]. Thus, we reviewed the relevant associated genes and summarized them (Table S3) [4]. Then, we retrieved each transcript using the genome data viewer and checked the position of the *p*- or q-arm of the chromosome relative to the telomere of 57 genes whose mutations causing congenital heart disease (CHD) (Table S4). 

**Figure 3:**
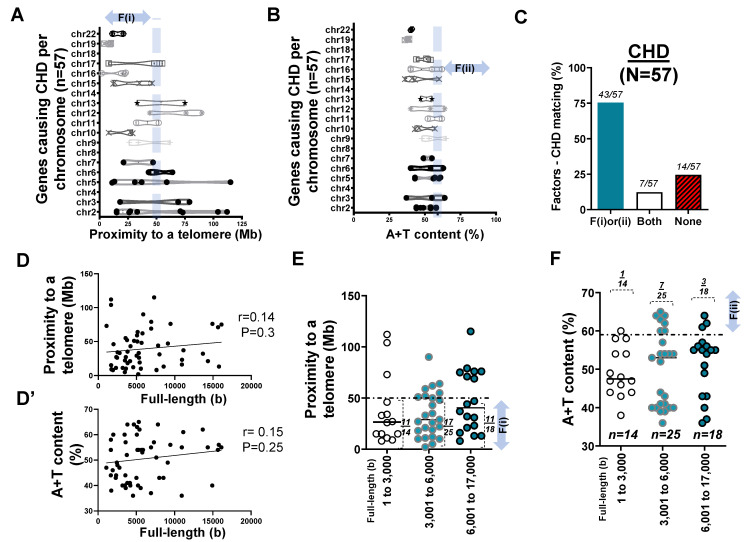
Genes (*n* = 57) whose mutations cause CHD. (**A**) Box and violin plots showing the distribution of the genes over chromosomes (chr) 2 to 22 with respect to proximity to the telomere. (**B**) Box and violin plots showing the distribution of the genes over chr 2 to 22 with respect to A+T content. (**C**) Bar graph demonstrating factors–CHD matching rate. ‘F(i)or(ii)’ represents the genes causing CHD satisfying either proximity to the telomere within 50 Mb or A+T content higher than 59%; ‘Both’ represents the genes meeting two factors alike. (**D**) Scatter plot showing the Pearson correlation between the full length of genes causing CHD and proximity to the telomere. (**D’**) Scatter plot demonstrating the Pearson correlation between the full length of genes causing CHD and A+T content. (**E**) Scatter plot showing 57 genes causing CHD with proximity to the telomere over full-length size of the gene. A horizontal dotted line indicates 50 Mb. (**F**) Scatter plot summarizing 57 genes causing CHD with A+T content over full-length size of the gene (unit of base or b). A horizontal dotted line indicates A+T content at 59%.

In assessing the list of genes causative of CHD, we found that a substantial number of listed genes were from mouse studies. After sorting out the duplicate genes appearing multiple times (Tables S4 and S6) [37], we investigated 57 human genes (Table S4) and surveyed F(i) for each gene (Figure 3). As we identified 57 gene loci (Table S5), we again observed that, in chromosomes of short physical length such as chromosome 19 and 22 (Figure 3A), causative genes were more likely to fulfill the F(i) as compared to those located in other chromosomes. 

To further test the hypothesis regarding F(ii), we obtained the A+T content of genes whose mutations were reported to cause CHD. Although causal genes were widely distributed in almost all human chromosomes, a smaller number of genes met F(ii) (Figure 3B). In assessing the factor–disease matching rate, >75% of genes causative of CHD satisfied the F(i) (*n* = 39/57) or F(ii) (*n* = 11/57) conditions. Furthermore, 7 of 57 known genes met both F(i) and F(ii), while ~25% (14 of 57 genes) belonged to neither F(i) nor F(ii) (Figure 3C). This supported the idea that if there was a disease-specific matching of the previously proposed two factors predicting genetic disorders of the heart, telomere proximity or A+T content might be able to predict CHD at a >75% match.

During the survey of nucleotide base pairs of the 57 genes, data entries suggested that the full-length size of a gene, if larger than 6000 bp, might indicate a high A+T content at >59%. Although a similar trend (upwards or increasing) was detected, the Pearson coefficient (r = 0.14) suggested that there was no significant correlation (*p* = 0.3) between the full-length size and F(i) in CHD. Intriguingly, the same was true for the relationship between the full-length size and F(ii) (r = 0.15; *p* = 0.25) (Figure 3D,D’).

We then organized F(i) and F(ii) with respect to the base pair size of each gene. We grouped genes into three categories: 1–3000 bp (*n* = 14), 3001–6000 bp (*n* = 25), and 6001–17,000 bp (*n* = 18) (Table S4; Figure 3E,F). There was no significant correlation in F(i) with respect to the gene full-length size (*p* = 0.3) (Figure 3E). Unlike the previous report [4], however, pairwise comparisons using Tukey’s post hoc test after one-way ANOVA suggested that there was no significant correlation (*p* = 0.75) in F(ii) with respect to the full-length size (bp) between the groups (Figure 3F). 

### 2.4. Mapping of 27 Genes Associated with TAA

**Figure 4:**
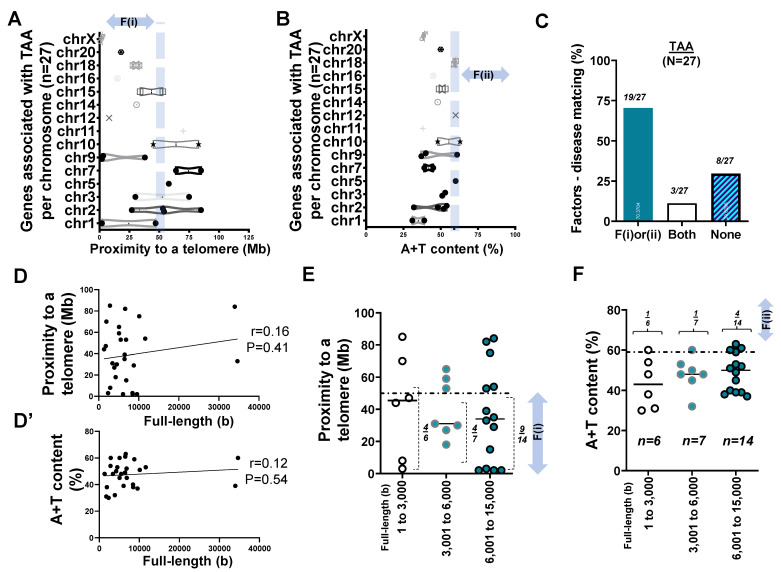
Genes (*n* = 27) associated with thoracic aortic aneurism (TAA) (**A**) Box and violin plots displaying the distribution of genes susceptible to TAA with respect to proximity to telomeres over chromosomes (chr) 1 to X. (**B**) Box and violin plots summarizing the full distribution of genes susceptible to TAA with respect to A+T content over chr 1 to X. (**C**) Bar graph demonstrating factors–TAA matching rate. (**D**) Scatter plot exhibiting the Pearson correlation between the full length of genes susceptible to TAA and their proximity to telomeres. (**D’**) Scatter plot showing the Pearson correlation between the full length (base or b) of genes associated with TAA and A+T content. (**E**) Scatter plot showing proximity to telomeres of 27 genes susceptible to TAA with respect to three subgroups of the gene size by full length in base or b. A horizontal dotted line indicates 50 Mb. (**F**) Scatter plot summarizing A+T content of genes susceptible to TAA with respect to the gene size in base or b. A horizontal dotted line indicates 59%. The statistical difference after one-way ANOVA among three groups of 1 to 3000, 3001 to 6000, and 6001 to 15,000 at *p* > 0.05 (**A**, **B**, **E**, and **F**).

Using a compilation of updated literature on TAA [38,39,40], we conducted our analysis according to the literature selection criteria set forth previously in the methods [39]. We checked the position of the *p*- or q-arm of the chromosome relative to the telomere of 27 genes associated with TAA (Table S5). In assessing the list of genes in Table S5, we surveyed the distance between each gene locus and its telomere using the F(i) (Figure 4A). As we identified 27 gene loci listed in Table S5, it was evident that, in chromosomes of short physical length (e.g., chr 18–20), the “distance to a telomere” was within 50 Mb, thereby fulfilling F(i). 

To further test our hypothesis, we obtained the A+T content of genes associated with TAA. Unlike CHD and other genetic disorders [4], genes associated with TAA rarely met high A+T content at >59% (Figure 4B). When we examined two factors in 27 genes of TAA, <66% satisfied the proximity to telomeres (*n* = 17/27) or high A+T content (*n* = 5/27) conditions (collectively 70% for two factors). Evidently, 3 of 27 known genes met both F(i) and F(ii), while ~28% (8 of 27 genes) belonged to neither F(i) nor F(ii). This suggests that if there is a disease-specific matching of the previously proposed two factors, telomere proximity or A+T content can explain TAA at 70% match (19 of 27) (Figure 4C). 

During the survey of nucleotide base pairs of the 27 genes, data entries suggested that the full-length size of a gene, if larger than 6,000 bp, might lead to the said gene having high A+T content at >59%. Although a similar trend was found that was consistent with the recent report [4], the Pearson coefficient (r = 0.16) suggested that there was no significant correlation (*p* = 0.41) between the full-length size and proximity to telomeres in TAA. The same was true for the relationship between the full-length size and A+T content (r = 0.12; *p* = 0.54) (Figure 4D,D’).

We then organized F(i) and F(ii) with respect to the base pair of each gene. Consistent with the previous analysis (Figure 1, Figure 2 and Figure 3), we grouped genes into three categories: 1–3000 bp (*n* = 6), 3001–6000 bp (*n* = 7), and 6001– 15,000 bp (*n* = 14) (Table S5). Statistical analysis suggested that there was no significant difference in F(i) with respect to the gene full-length size (*p* = 0.87). Furthermore, pairwise comparisons using Tukey’s post hoc test after one-way ANOVA suggested that there was no significant difference (*p* = 0.5) in F(ii) with respect to the full-length size (bp) between the groups (Figure 4E,F).

## 3. Discussion

Our review of idiopathic hypertension or EH [41] resulted in a challenging profile of hypertension candidate genes at the q-arm of chromosome 1. The idea that a poor match indicated that the disease (EH) was multigenic—while a higher match implied the disease was monogenic—needed to be further verified. Hence, we investigated the 79 loci associated with MH and contributing to the genetic architecture of hypertension [35], demonstrating that genetic loci listed in this recent study [35] harbor a proximity to telomeres and nucleotide compositions (a ~81% match) similar to those 108 genes causative of congenital disease of the CNS. These two factors matched with the disease at ~90%, along with the significant correlation of the nucleotide size and A+T content (Table 1) [4]. Even as the literature [37] we sought for testing our hypothesis depicted a unique linkage from the causal genes to the congenital heart defect or CHD through cilia or ciliopathy-related loci (Table S4) [4], the chromosomal characteristics of 57 genes causative of CHD (Figure 3) showed a similar feature (75% match) to genes proposed in MH (81% match) rather than EH (53% match). 

A factor–disease matching rate higher than 80% indicates a likely correlation between these factors and the mutations in a particular gene causative of the disease. In the case of genetic hypertension or MH, 81% of examined genes satisfied either proximity to telomeres or A+T content at >59%. This seems to indicate that the development of hypertension can be directly influenced by mutations of these specific genes. Additionally, this high factor–disease matching rate could provide a reliable method in the future of predicting an individual’s risk of developing MH, at least for >25 rare mutations claimed by the authors [35], through the examination of the family history and genetic mutations of an individual.

On the other hand, if the factor–disease matching rate is closer to 50%—such as is the case with EH—this seems to indicate a much more complex relationship between the disease and its associated genes. Specifically, it is likely that this low matching rate is indicative of the disease being multigenic in nature. Indeed, EH has been previously identified as idiopathic in nature, meaning that a variety of factors impact its development. While proximity to telomeres and A+T content can serve as an accurate predictive measure for diseases that can be induced by a mutation in a single gene, it will inherently be much less accurate at predicting diseases whose causes are not fully understood and documented. Thus, this supports the idea that the factor–disease matching rate is much lower for this idiopathic disease.

While the genes involved with MH, CHD, and TAA appear to be relatively long in terms of base pair length, the genes involved with CH, AD, and fPD appear to be much shorter in length. This significant difference in nucleotide length—yet relative similarity as to factor–disease matching rates—potentially indicates the specific factor evoking mutations (and therefore likely the type of mutation taking place) in each of these genes. Since genes associated with diseases of the cardiovascular system are comparatively longer compared to those associated with the CNS, it is potentially more likely that these genes have a greater A+T content (>59%), on account of their greater nucleotide length. At the same time, a longer gene is less likely to be located (at least entirely) within 50 Mb of the telomere. While a shorter gene might easily fall within 50 Mb of the telomere, a much longer gene might fall partially or entirely outside this proximity. This could result in a difference of factor–disease matching rate—at least in terms of which factor(s) matched which disease—between CVD and CNS disease. Based on the contents of Table 1, it is tempting to argue that the genes associated with the CNS—since they are both comparatively short in nucleotide full length (FL) and have significant FL-A+T correlation—are more likely to meet both F(i) and F(ii), while the genes associated with CVD potentially only meet one of the two factors. 

In the previous studies on human chromosomes, two factors associated with high mutation rate [4,12] were proposed: (i) the location within 50 Mb from the chromosome end [12,42] and (ii) A+T content higher than average (59%) [12]. Telomeres by themselves are considered to be a mechanism to protect chromosomes, since cells do not tolerate the presence of unprotected chromosome ends [43]. Interestingly, chromosomal DNAs adjacent to telomeres are vulnerable to DNA damage or mutagenesis [12], partly because of telomere dysfunction in telomerase null conditions [44]. It is also noted that genes in close proximity to telomeres are silenced due to the repressive nature of specific telomere-binding proteins such as heterochromatin [32]. The degree of repression declines with distance from the telomere, limited to ~100 kb [45]. While a plethora of convincing evidence exists for the first factor, the high mutation rate associated with A+T rich regions in human chromosomes [12] has been too complex [10,11] to summarize until the chimpanzee and human genomes were successfully sequenced [8,9]. 

We demonstrated the utility of two factors, which were narrowed down from more than ten factors previously proposed [12]. The strengths of this study lie in the accuracy of predictability at >80% in CVDs and >90% in a genetic disease of the CNS such as congenital hydrocephalus [4] by using only two factors: proximity to telomeres and DNA compositions of the human disease gene. The limitations, likewise, are those human diseases caused by genetic mutation(s) as well as environmental factors such as sedentary lifestyle, unhealthy dietary intake, and exposures to toxins playing a complex role, in which the matching rate between the factors and the disease is only at ~50%.

The clinical utility of the present study is that clinicians can screen causative gene mutations found in preclinical or animal models in comparison with human chromosome analysis summarized in this study using a genome data viewer (NIH software) and GC content calculator (open software). The physical length of several human chromosomes can also be noted to save time in applying the first factor. A mouse causative gene in mouse chromosome 1 does not always locate in the same chromosome number of the human genomes. Therefore, species difference matters in physical length/proximity measure between a gene and its telomere in humans vs. animals. For example, *L1CAM* gene, well known in hydrocephalus research, is in chromosome X of humans and mice. *MYC* gene, however, is in human chromosome 8, while in mouse, it is in chromosome 15. The physical length of chromosome 8 (50 mm) vs. chromosome 15 (35 mm) differs, resulting in a different potential to meet the first factor. It should also be noted that mouse chromosomes do not have *p*- or *q*-arm. 

Our analysis presented in this study suggests that these two factors can explain the cause of genetic mutations in 79 loci proposed in MH [35] roughly at an 80% rate. In comparison, these two factors proposed can only explain the cause of idiopathic diseases such as EH [41] at a 53% rate. The proposed genomic analyses demonstrate an intermediate matching rate or a mediocre predictability (~75% or less) between the cause of genetic mutations and the disease in the cases of CHD, aortic aneurysm (TAA), and age-related degenerative disorders [4], which warrant further investigations on the establishment of missing links.

## 4. Materials and Methods

### 4.1. Database 

The literature survey was carried out with emphasis on diseases reported to be associated with vascular stiffening: congenital heart disease (CHD) [19], thoracic aortic aneurysm (TAA) [20,21], and hypertension [17,18]. We utilized the PubMed database, as well as Google Scholar, in order to select the source literature containing candidate genes or loci with mutations to be analyzed regarding each disease. Combinations of a single keyword and the disease name were attempted. If we were unable to locate relevant literature through the use of a single keyword, a second keyword was added. 

### 4.2. Keywords in Literature Search

**Figure 5:**
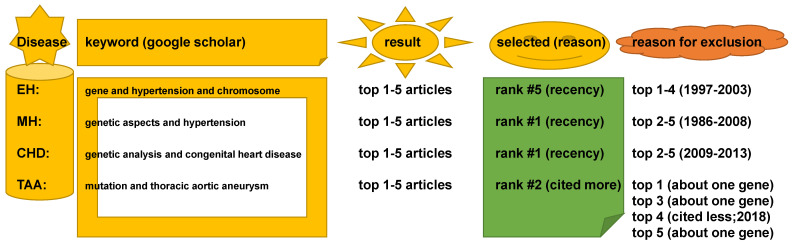
A schematic representation of the searching strategy for literature used in the genetic analysis of this study: EH, essential hypertension, MH, monogenic hypertension, CHD, congenital hypertension, TAA, thoracic aortic aneurysm.

The five words ‘gene’, ‘genetic’, ‘mutation’, ‘analysis’, and ‘chromosome’ were used with the disease name in Google Scholar. For example, the search was done using ‘(a keyword) and (the disease)’. The search was considered successful if multiple genes or genetic mutations were clearly tabulated as a causal and/or associated locus in the literature, consistent with the recent report [4]. The second search was conducted with a refined keyword if no relevant literature was originally located. As a result, genes associated with EH were found with keywords ‘gene’ and ‘hypertension’ and ‘chromosome’. Genes associated with MH were found with keywords, ‘genetic mutation’ and ‘hypertension’. Genes associated with CHD were identified with keywords, ‘genetic analysis’ and ‘congenital heart disease’. Genes related to TAA were searched for with keywords ‘mutation’ and ‘thoracic aortic aneurysm’. For each disease, one pertinent piece of literature was selected among the top 5 results sorted by relevance (to the search keyword in Google Scholar) and by recency (the most recent paper among the top 5). The paper was excluded if the study pertained only to a single gene. Between two articles from the same group/authors, the more cited article was selected in the present study (Figure 5).

### 4.3. Open-Source Software

We utilized the publicly open Genome Data Viewer, version 5.1, (https://www.ncbi.nlm.nih.gov/genome/gdv/ accessed on 9 May 2021; NIH National Center for Biotechnology Information, Bethesda, MD) and GC content calculator (Biologics International Corp, Indianapolis, IN, USA) to obtain A+T content (https://www.biologicscorp.com/tools/GCContent/#.XvctCi-z2uV, accessed on 9 May 2021) [4]. 

Approximation of proximity to a telomere: 

The biological basis for the apparently high mutation rate in human chromosomes has been previously described. We followed the previous method in the approximation of proximity to a telomere [4]. As a result, in this study, we calculated the nucleotide compositions of the gene and focused on the position of the gene and its distal end locus of each arm (telomere) with the following premise:

(1) If recombination frequency [42] is less than (≤) 50 cM, genes are linked;
(2) if recombination frequency is higher than 50 cM, genes are not linked,

where 1 centimorgan (cM) ≅ 1 million base pair (Mb) [46].

### 4.4. Data Plot and Statistical Methods

Prism was used to plot a bar graph and box and violin plot of the data obtained during analysis with the genome data viewer. Statistical analyses were performed using Prism (version 8, GraphPad Software Inc.). Normal distribution of the data was confirmed using the Shapiro–Wilk normality test (α < 0.05). A two-sided unpaired *t*-test was used for comparison of two different groups, unless stated differently. Tukey’s multiple comparisons test following one-way analysis of variance was used for comparison of more than two groups. The difference between data sets was considered significant at *p* < 0.05; *p* values are identified in the figures and legends as * *p* < 0.05, ** *p* < 0.01, *** *p* < 0.005.

## 5. Conclusions

- Two factors reasonably explain the genetic mutations of cardiovascular disorders such as MH (>25 rare mutations) and the genetic architecture of hypertension (53 SNPs) at an 81% matching rate.
- Our result using the first factor—particularly in MH and the genetic architecture of hypertension—contributed by genetic variants suggests that susceptible genes located in chromosomes 18 to 22 more likely meet proximity to telomeres (<50 Mb) due to their short chromosomal lengths.
- Factor–nucleotide size relationships suggest that the full-length size of a gene associated with MH and the genetic architecture of hypertension longer than 6000 bp likely show high A+T content at >59%.
- If genes susceptible to CVD with mutations do not satisfy two criteria or the factors according to the previous suggestion [12], then neutral or deleterious mutations may appear in a region that is unlikely to be mutated, or the methods by which susceptible genes are filtered out (e.g., microsatellite markers vs. next generation sequencing) should be verified, or the contribution of environmental factors (e.g., high salt diet, body mass index, and sedentary lifestyle) might be more significant than that of genetic factors.

## Figures and Tables

**Table 1:** Characteristics of genes associated with CVD and CNS disorders by two factors.

**Cardiovascular System**	**MH** [35]	**CHD** [37]	**TAA** [39]	**EH** [41]
Factor–disease match	81%	75%	70%	53%
No. of genes (loci) FL–A+T correlation Short–Mid–Long (−3000)(−6000)(−17,000 bp) Relative type of gene size	*n* = 79 sig. 22:29:28 (1:1.3:1.3) longer	*n* = 57 NS 14:25:18 (1:1.8:1.3) longer	*n* = 27 NS 6:7:14 (1:1.2:2.3) longer	*n* = 41 NS 16:15:10 (1.6:1.5:1) shorter
**CNS**	**CH [4]**	**AD [4]**	**fPD [4]**	
Factor–disease match	>90%	84%	59%	
No. of genes (loci) FL–A+T correlation Short–Mid–Long (−3000)(−6000)(−15,000 bp) Relative type of gene size	*n* = 108 sig. 40:39:29 (1.4:1.3:1) shorter	*n* = 70 sig. 39:22:9 (4.3:2.4:1) shorter	*n* = 17 sig. 7:5:5 (1.4:1:1) shorter	

CVD: cardiovascular disease; CNS: central nervous system; MH: monogenic or Mendelian hypertension; CHD: congenital heart disease; TAA: thoracic aortic aneurysm; EH: essential hypertension; Sig.: significant; NS: not significant; FL: full-length (size of a gene); A+T: adenine and thymine content; CH: congenital hydrocephalus; AD: Alzheimer’s disease; fPD: familial Parkinson’s disease.

## Data Availability

The data presented in this study are available in supplementary material as Table S1 through Table S6.

## Supplementary Materials

The following are available online at https://www.mdpi.com/article/10.3390/ijms22105057/s1, Table S1: Proximity to telomeres vs. A+T content in candidate genes of essential hypertension, Table S2: Survey of factors in genes near the loci associated with hypertension, Table S3: An outline of genes causing congenital heart disease (CHD), Table S4: Proximity to telomeres vs. A+T content in genes causing congenital heart disease. Table S5: Genes associated with syndromic and non-syndromic thoracic aortic aneurysm, Table S6: Mouse CHD genes and associated human diseases.
