# Supplementary material for: Factors Associated with Mutations: Their Matching Rates to Cardiovascular and Neurological Diseases
Source: Int J Mol Sci. 2021 May 11;22(10):5057. doi: 10.3390/ijms22105057 (PMC8151074; doi:10.3390/ijms22105057)
Supplement: Supplementary file 1 [file ijms-22-05057-s001.zip › ijms-1185208-supplementary.pdf]

Supplementary Materials for

Factors associated with mutations: Their matching rates to cardiovascular and neurological diseases

Hannah B. Lucas [1], Ian McKnight [1], Regan Raines [1], Abdullah Hijazi [1], Christoph Hart [1], Chan Lee [2], Do-Gyoon Kim [3], Wei Li [4], Peter H.U. Lee [5,6], Joon W. Shim [1,*]

Correspondence to: shim@marshall.edu

This file includes:

Tables S1 to S6

Table S1. Proximity to telomeres vs. A+T content in candidate genes of essential hypertension

gene ID	chr	gene locus near at	telomere locus near at	gene to telomere (Mbp); <50cM**?	A+T %	A+T >59%?	FL (bp)	rationale [REF]
ATP1A2	1	160 Mb	247 Mb	247-160=87	100-53=47	47	5435	1
ATP1A4	1	160	247	247-160=87	100-53=47	47	3809	1
RGS4	1	163	247	247-163=84	100-41=59	59	2984	1
PDE2A	11	72	135	135-72=63	100-58=42	42	4284	1
RGS5	1	163	247	247-163=84	100-36=64	64	5670	1
SAC	1	167	247	247-167=80	100-44=56	56	5342	1
RE2	1	168	247	247-168=79	100-51=49	49	8437	1
ADAMTS6	5	65	180	180-65=115	100-43=57	57	7309	1
ATP1B1	1	169	247	247-169=78	100-42=58	58	2216	1
SLC19A2	1	169	247	247-169=78	100-41=59	59	3612	1
SELP	1	169	247	247-169=78	100-50=50	50	3157	1
SELL	1	169	247	247-169=78	100-44=56	56	2358	1
SELE	1	169	247	247-169=78	100-42=58	58	3875	1
GPR52	1	174	247	247-174=73	100-43=57	57	1582	1
ANGPTL1	1	178	247	247-178=69	100-36=64	64	3543	1
SOAT1	1	179	247	247-179=68	100-40=60	60	6840	1
NPHS2	1	179	247	247-179=68	100-52=48	48	1870	1
CACNA1E	1	181	247	247-181=66	100-49=51	51	16291	1
RGS16	1	182	247	247-182=65	100-53=47	47	2408	1
RGS8	1	182	247	247-182=65	100-50=50	50	5858	1
RGS18	1	192	247	247-192=55	100-33=67	67	2145	1
RGS1	1	192	247	247-192=55	100-38=62	62	1352	1
RGS13	1	192	247	247-192=55	100-32=68	68	1554	1
RGS2	1	192	247	247-192=55	100-42=58	58	1348	1
SLICK	1	196	247	247-196=51	100-35=65	65	5984	1
PTPRC	1	198	247	247-198=49	100-38=62	62	5357	1
GPR25	1	200	247	247-200=47	100-73=27	27	1198	1
CACNA1S	1	201	247	247-201=46	100-56=44	44	6028	1
TNNT2	1	201	247	247-201=46	100-56=44	44	1156	1

Table S1. Continued

gene ID	Chr	gene locus near at	telomere locus near at	gene to telomere (Mbp); <50cM**?	A+T %	A+T >59%?	FL (bp)	rationale [REF]
TNNI1	1	201Mb	249Mb	249-201=48	100-47=53	53	6110	1
ET(B)R-LP-2	1	202 Mb	249Mb	249-202=47	100-43=57	57	6529	1
PTPN7	1	202 Mb	249 Mb	249-202=47	100-45=55	55	2783	1
PPP1R12B	1	202 Mb	249 Mb	249-202=47	100-53=47	47	15244	1
ADORA1	1	203 Mb	249 Mb	249-203=46	100-49=51	64	2901	1
ATP2B4	1	203Mb	249Mb	249-203=46	100-54=46	46	8697	1
REN	1	204 Mb	249 Mb	249-204=45	100-46=54	54	1462	1
PPP1R15B	1	204 Mb	249Mb	249-204=45	100-58=42	42	5259	1
SLC41A1	1	205 Mb	249Mb	249-205=44	100-48=52	52	5017	1
SLC26A9	1	206 Mb	249 Mb	249-206=43	100-47=53	53	4791	1
CAMK1G	1	209 Mb	249 Mb	249-209=40	100-47=53	53	2456	1

Table S1. Continued

gene ID	chr	gene locus near at	telomere locus near at	gene to telomere (Mbp); <50cM**?	A+T %	A+T >59%?	FL (bp)	rationale [REF]
RGS4	1	163 Mb	249 Mb	249-163=86	100-(40.7)41=59	59	3875	1
RGS5	1	163 Mb	249 Mb	249-163=86	100-36=64	64	722	1
ATP1B1	1	169 Mb	249 Mb	249-169=80	100-42=	58	2216	1
F5	1	170 Mb	249 Mb	249-170=79	100-43=57	57	9132	1
SELP	1	170 Mb	249 Mb	249-170=79	100-50=50	50	3157	1
SELL	1	170 Mb	249 Mb	249-170= 79	100-44=56	56	2358	1
SELE	1	170 Mb	249 Mb	249-170= 79	100-42=58	58	3875	1
NPHS2	1	180 Mb	249 Mb	249-180=69	100-52=48	48	1870	1
ADORA1	1	203 Mb	249 Mb	249-203=46	100-62=38	38	2901	1
REN	1	204 Mb	249 Mb	249-204=45	100-55=45	45	1462	1

Table S2. Survey of factors in genes near the loci associated with hypertension[2]

gene ID	chr	gene locus near at	telomere locus near at	gene to telomere (Mbp); <50cM**?	A+T %	A+T >59%?	FL (bp)	rationale [access #]
CLCNKB	1	16Mb	0 Mb	16-0=16	18, 24	42	2562	Nm_000085
SDHB	1	17Mb	0 Mb	17-0=17	29,26	55	1015	Nm_003000
CASZ1	1	10Mb	0 Mb	10-0=10	20,18	38	4403	Nm_017766
MTHFR, CLCN6, NPPA, NPPB	1	11Mb	0 Mb	11-0=11	21,23	44	7018	Nm_005957
ATP1A1	1	116Mb	0 Mb	116-0=116	24,26	50	3670	Nm_000701
SDHC	2	161Mb	248 Mb	248-161=87	21,33	54	1308	Nm_003001
SLC16A1	2	112Mb	248 Mb	248-112=136	28,33	61	3753	Nm_003051
MDM4	2	204Mb	245 Mb	245-204=41	26,32	58	10050	Nm_002393
AGT	1	230Mb	247 Mb	247-230=17	22,24	46	2148	Nm_001382817
SLC4A5	2	74 Mb	0 Mb	74-0=74	23,23	46	3449	Nm_021196
FIGN	2	163 Mb	241 Mb	241-163=78	29,33	62	9537	Nm_018086
PDE1A	2	182 Mb	241 Mb	241-182=59	31,33	64	4829	Nm_005019
CUL3	2	224 Mb	241 Mb	241-224=17	31,32	63	6756	Nm_003590
VHL	3	10 Mb	0	10-0=10	25,30	55	4414	Nm_000551
HRH1	3	11 Mb	0	11-0=11	27,28	55	4427	Nm_000861
SLC4A7	3	27 Mb	0	27-0=27	30,36	66	7781	Nm_003615
MAP4, smarcc1	3	47 Mb	0	47-0=47	25,23	48	5891	Nm_002375
CACNA1D	3	53 Mb	0	53-0=53	26,26	52	9429	Nm_000720
Ulk4, ctnnb1	3	41 Mb	0	41-0=41	27,29	56	4294	Nm_017886
Mecom	3	169Mb	197 Mb	197-169=28	31,27	58	5462	Nm_004991
pdgfra	4	54 Mb	189 Mb	189-54=135	29,29	58	6378	Nm_006206
Fgf5	4	80 Mb	189 Mb	189-80=109	30,35	65	5313	Nm_004464
Slc39a8	4	102 Mb	189 Mb	189-102=87	27,31	56	3309	Nm_022154
Enpep, pitx2	4	110 Mb	189 Mb	189-110=79	31,32	63	6861	Nm_001977
Nr3c2	4	148 Mb	189 Mb	189-148=41	28,29	57	5792	Nm_000901
Gucy1a3,gucy1b3	4	155 Mb	189 Mb	189-155=34	31,32	63	9299	Nm_000856

Table S2. Continued: [2]

gene ID	chr	gene locus near at	telomere locus near at	gene to telomere (Mbp); <50cM**?	A+T %	A+T >59%?	FL (bp)	rationale [access #]
Sdha	5	0.21 Mb	0	0-0.21=0.21	23,26	49	2693	Nm_004168
Npr3	5	32 Mb	0	32-0=32	28,31	59	7454	Nm_000908
Klhl3	5	137 Mb	181	181-137=44	24,29	53	6805	Nm_017415
Ebf1	5	159 Mb	181	181-159=22	29,30	59	5405	Nm_024007
Hfe	6	26 Mb	0	26-0=26	27,32	59	5176	Nm_000410
Ddah2, hspa1l	6	31 Mb	0	31-0=31	20,19	39	1332	Nm_013974
Hla-dqb1	6	32 Mb	0	32-0=32	23,27	50	1605	Nm_002123
Cdk6	7	92 Mb	158 Mb	158-92=66	28,33	61	11654	Nm_001259
Pik3cg	7	106 Mb	158 Mb	158-106=52	29,30	59	7218	Nm_002649
Abp1, kcnh2	7	44 Mb	44 Mb	44 – 44 =0	30,22	52	3789	Nm_018051
enpp2	8	119 Mb	144 Mb	144-119=25	29,29	58	3242	Nm_006209
Cyp11b1	8	142 Mb	144 Mb	144-142=2	22,24	46	3540	Nm_000497
Cacnb2	10	18 Mb	0 Mb	18-0=18	30,30	60	6199	Nm_000724
Ret	10	43 Mb	133 Mb	133-43=90	20,22	42	4269	Nm_020630
C10orf107	10	61 Mb	133 Mb	133-61=72	20,22	42	1717	Nm_173554
VCL	10	74 Mb	133 Mb	133-74=59	27,26	53	6487	Nm_003373
Plce1	10	94 Mb	133 Mb	133-94=39	30,27	57	12481	Nm_016341
Cyp17a1	10	102 Mb	133 Mb	133-102=31	24,25	49	1750	Nm_000102
Adrb1	10	114 Mb	133 Mb	133-114=19	18,24	42	3039	Nm_000684
H19	11	1 Mb	0 Mb	1-0=1	20,18	38	2362	Nr_002196
Lsp1	11	1 Mb	0 Mb	1-0=1	22,17	39	1585	Nm_002339
Adm	11	10 Mb	0	10-0=10	22,24	46	1492	Nm_001124
Sox6	11	16 Mb	0	16-0=16	29,30	59	8975	Nm_017508
Plekha7	11	16 Mb	0	16-0=16	24,22	46	4984	Nm_175058
Pik3c2a	11	17 Mb	0	17-0=17	32,32	64	8428	Nm_002645
Map3k11	11	65 Mb	134 Mb	134-65=69	17,17	34	3543	Nm_002419
Flj32810	11	100 Mb	134 Mb	134-100=34	30,31	61	8156	Nm_152432
Sdhaf2	11	61 Mb	134 Mb	134-61=73	26,25	51	1186	Nm_017841

Table S2. Continued: [2]

gene ID	chr	gene locus near at	telomere locus near at	gene to telomere (Mbp); <50cM**?	A+T %	A+T >59%?	FL (bp)	rationale [access #]
Sdhd	11	112 Mb	134 Mb	134-112=22	27,34	60	1339	Nm_003002
Adamts8	11	130 Mb	134 Mb	134-130=4	19,19	38	3628	Nm_007037
Kcnj1	11	128 Mb	134 Mb	134-128=6	29,30	59	2333	Nm_000220
Kcnj5	11	128 Mb	134 Mb	134-128=6	23,23	46	6068	Nm_000890
Wnk1	12	0	0	0	26,25	51	10052	Nm_014823
Hox3c	12	54 Mb	132 Mb	132-54=78	25,24	49	1651	Nm_004503
Atp2b1	12	89 Mb	132 Mb	132-89=43	31,33	62	6967	Nm_001682
Aldh2	12	111 Mb	132 Mb	132-111=21	27,26	53	9561	Nm_000690
Tbx5	12	114 Mb	132 Mb	132-114=18	28,25	53	3921	Nm_000192
Slc12a1	15	48 Mb	101 Mb	101 – 48 =53	30,31	61	4708	Nm_000338
Cyp1a1	15	74 Mb	101 Mb	101-74=27	23,25	48	2600	Nm_000499
fes	15	90 Mb	101 Mb	101-90=11	20,20	40	2737	Nm_002005
Scnn1b	16	23 Mb	0	23-0=23	21,21	42	2560	Nm_000336
Umod	16	20 Mb	0	20-0=20	20,23	43	2315	Nm_003361
Slc12a3	16	56 Mb	90 Mb	90-56=34	23,24	47	5567	Nm_000339
Hsd11b2	16	67 Mb	90 Mb	90-67=23	17,21	38	1896	Nm_000196
Nfat5	16	69 Mb	90 Mb	90 – 69 = 21	28,33	61	13235	Nm_006599
Wnk4	17	42 Mb	83 Mb	83 – 42=41	19,21	40	4199	Nm_032387
Gosr2	17	46 Mb	83 Mb	83-46=37	23,30	53	3932	Nm_004287
Znf652	17	49 Mb	83 Mb	83-49=34	28,34	62	11347	Nm_014897
Prkca	17	66 Mb	83 Mb	83-66=17	25,27	53	8964	Nm_002737
Nedd4l	18	58 Mb	79 Mb	79-58=21	27,29	56	8573	Nm_015277
Jag1	20	10 Mb	0	10-0=10	25,27	52	5940	Nm_000214
Gnas	20	58 Mb	64 Mb	64-58=6	25,20	45	1854	Nm_000516
Atp2b3	X	153 Mb	155 Mb	2	23,22	45	6928	Nm_021949

Table S3 An outline of genes causing congenital heart disease (CHD)

#	Gene	Protein	key functions	mutant phenotype	REF
1	ADAMTS6	a disintegrin and metalloproteinase with thrombospondin motifs 6	presenting undiscovered pathogenic mutations responsible for novel Mendelian disorders	Congenital heart disease	3
2	Anks6	ANKYRIN REPEAT AND STERILE ALPHA MOTIF DOMAINS-CONTAINING PROTEIN 6	assembles a complex of ciliary proteins required for renal and cardiovascular development	renal phenotype	https://www.omim.org/entry/615370
3	Ap1b1	ADAPTOR-RELATED PROTEIN COMPLEX 1, BETA-1 SUBUNIT	that GGAs and the AP-1 complex interact to package mannose 6-phosphate receptors into AP-1-containing coated vesicles.	Keratitis-ichthyosis-deafness syndrome, autosomal recessive	https://www.omim.org/entry/600157
4	Ap2b1	ADAPTOR-RELATED PROTEIN COMPLEX 2, BETA-1 SUBUNIT	The AP2B1 protein is part of the AP2 coat assembly protein complex (see 601024) and links clathrin (118960) to receptors in the coated vesicles	cargo selection and vesicle formation	https://www.uniprot.org/uniprot/P63010 https://www.omim.org/entry/601025
5	ARMC4	ARMADILLO REPEAT-CONTAINING PROTEIN 4	encodes a protein required for a late step in proper targeting and anchoring of outer dynein arms	Ciliary dyskinesia	https://www.omim.org/entry/615408
6	Cc2d2a	COILED-COIL AND C2 DOMAINS-CONTAINING PROTEIN 2A	component of a protein complex in the basal body, a ring-like structure that functions in the transition zone at the base of cilia	COACH syndrome<br>Joubert syndrome 9<br>Meckel syndrome 6	https://www.omim.org/entry/612013
7	CCDC39	COILED-COIL DOMAIN-CONTAINING PROTEIN 39	ocalizes to ciliary axonemes and is essential for assembly of inner dynein arms and the dynein regulatory complex	Ciliary dyskinesia	https://www.omim.org/entry/613798
8	Cep110	Centrosomal Protein Of 110 KDa	involved in cell cycle progression and cytokinesis. During the late steps of cytokinesis, anchors exocyst and SNARE complexes at the midbody, thereby allowing secretory vesicle-mediated abscission.	Eczema<br>Abnormality of refraction<br>atopic asthma<br>acute myeloid leukemia<br>rheumatoid arthritis	https://www.genecards.org/cgi-bin/carddisp.pl?gene=CNTRL

Table S3 (continued)

#	Gene	protein	key functions	mutant phenotype	REF
9	Cep110	Centrosomal Protein Of 110 KDa	involved in cell cycle progression and cytokinesis. During the late steps of cytokinesis, anchors exocyst and SNARE complexes at the midbody, thereby allowing secretory vesicle-mediated abscission.	Eczema<br>Abnormality of refraction<br>atopic asthma<br>acute myeloid leukemia<br>rheumatoid arthritis	https://www.genecards.org/cgi-bin/carddisp.pl?gene=CNTRL
10	CEP290	CENTROSOMAL PROTEIN, 290-KD	The CEP290 gene encodes a centrosomal protein involved in ciliary assembly and ciliary trafficking	Senior-Loken syndrome 6<br>Meckel syndrome 4<br>Leber congenital amaurosis 10<br>Joubert syndrome 5<br>Bardet-Biedl syndrome 14	https://www.omim.org/entry/610142?search=CEP290
11	Cfc1	CRYPTIC PROTEIN	play key roles in intercellular signaling pathways during vertebrate embryogenesis	Heterotaxy, visceral, 2, autosomal	https://www.omim.org/entry/605194
12	Cml5/nat8	N-ACETYLTRANSFERASE 8;	catalyzes the last step of mercapturic acid formation by acetylating cysteine S-conjugates to mercapturic acids	CHD	https://www.genecards.org/cgi-bin/carddisp.pl?gene=NAT8
13	Cxcr4	CHEMOKINE, CXC MOTIF, RECEPTOR 4;	encodes a CXC chemokine receptor specific for stromal cell-derived factor-1	Myelokathexis, isolated<br>WHIM syndrome	https://www.genecards.org/cgi-bin/carddisp.pl?gene=CXCR4<br>https://www.omim.org/entry/162643
14	Daw1	Dynein Assembly Factor With WD Repeats 1	a Protein Coding gene.	Ciliary Dyskinesia, Primary, 2<br><br>Dextro-Looped Transposition Of The Great Arteries.	https://www.genecards.org/cgi-bin/carddisp.pl?gene=DAW1
15	Dctn5	dynactin subunit 5	encodes a subunit of dynactin, a component of the cytoplasmic dynein motor machinery involved in minus-end-directed transport.	N/A	https://hive.biochemistry.gwu.edu/biomuta/branchview/Q9BTE1
16	DNAH11	DYNEIN, AXONEMAL, HEAVY CHAIN 11	a putative axonemal outer dynein arm heavy chain	Ciliary dyskinesia, primary, 7, with or without situs inversus	4

Table S3 (continued)

#	Gene	protein	key functions	mutant phenotype	REF
17	DNAH5	DYNEIN, AXONEMAL, HEAVY CHAIN 5	responsible for force production and ATPase activity and contain a highly conserved catalytic domain with 4 P-loop consensus motifs involved in nucleotide binding	Ciliary dyskinesia, primary, 3, with or without situs inversus	https://www.omim.org/entry/603335?search=DNAH5&highlight=dnah5
18	DnaI1	DYNEIN, AXONEMAL, INTERMEDIATE CHAIN 1;	Causes primary ciliary dyskinesia.	Ciliary dyskinesia, primary, 1, with or without situs inversus	https://www.omim.org/entry/604366?search=DnaI1&highlight=dnai1
19	Dnm2	DYNAMIN 2	DNM2 interacts tightly with actin and microtubule networks and may have a role in centrosome function	Centronuclear myopathy 1<br><br>Charcot-Marie-Tooth disease, axonal type 2M<br><br>Charcot-Marie-Tooth disease, dominant intermediate B<br><br>Lethal congenital contracture syndrome 5	https://www.omim.org/entry/602378?search=Dnm2&highlight=dnm2
21	Dync2h1	Dynein Cytoplasmic 2 Heavy Chain 1	Involved in retrograde transport in the cilium, has a role in intraflagellar transport, and is a process required for ciliary/flagellar assembly	Polydactyly, abnormal skeletogenesis, polycystic kidneys, and CHD	4
22	Dyx1c1	Dyslexia susceptibility 1 candidate gene 1 protein	**Required for axonemal dynein assembly and ciliary motility**	Primary ciliary dyskinesia (PCD), dyslexia, neuronal migration, and CHD	5
23	Foxj1 (m)	Forkhead box protein J1	A member of the Forkhead/winged helix (FOX) family of transcription factors that is involved in ciliogenesis and regulates the transcription of genes that control the production of motile cilia	Systemic lupus erythematosus, allergic rhinitis, breast cancer, clear renal cell carcinoma, Axenfeld–Rieger syndrome, hydrocephalus, and CHD	6
24	Frem2	FRAS1 Related Extracellular Matrix 2	Protein localizes to the basement membrane, forming a ternary complex that plays a role in epidermal-dermal interactions and is important for the integrity of skin and renal epithelia	Fraser syndrome and CHD	7
21	Dync2h1	Dynein Cytoplasmic 2 Heavy Chain 1	Involved in retrograde transport in the cilium, has a role in intraflagellar transport, and is a process required for ciliary/flagellar assembly	Polydactyly, abnormal skeletogenesis, polycystic kidneys, and CHD	4

Table S3 (continued)

#	Gene	protein	key functions	mutant phenotype	REF
25	Fuz	Fuzzy Planar Cell Polarity Protein	Involved in ciliogenesis and directional cell movement	Neural tube defects Alzheimer's, Parkinson's diseases, and CHD	8
26	Hectd1	E3 ubiquitin ligase Hectd1	Related pathways are Innate Immune Systemand Class I MHC mediated antigen processing and presentation is a protein coding gene	Embryonic lethality with abnormal placental development, exencephaly, and CHD	9
27	Ift140	Intraflagellar Transport 140 homolog	Involved in the process of intraflagellar transport (IFT), a process that is essential for the formation and maintenance of most eukaryotic cilia and flagella	Renal cystic disease, Mainzer Saldino Syndrome, nephronophthisis, Jeune Syndrome, Lebers congenital amaurosis, and CHD	10
28	Ift74	Intraflagellar Transport 74	Encodes a core intraflagellar transport (IFT) protein which belongs to a multi-protein complex involved in the transport of ciliary proteins along axonemal microtubules	Amyotrophic lateral sclerosis, frontotemporal dementia, Bardet-Biedl Syndrome, and CHD	11
29	Jbts17	Ciliogenesis And Planar Polarity Effector 1	Involved in the establishment of cell polarity required for directional cell migration.	Joubert syndrome, oral-facial-digital syndrome type VI, and CHD	12
30	Kif7	Kinesin Family Member 7	This protein plays a role in the sonic hedgehog (SHH) signaling pathway through the regulation of GLI transcription factors	Hydrolethalis, acrocallosal syndrome, Joubert syndrome, and CHD	13
31	Lox	Lysyl Oxidase	Catalyzes the conversion of lysine molecules into highly reactive aldehydes that form cross-links in extracellular matrix proteins	Thoracic aortic aneurysms and dissections, and CHD	14
32	Lrp1	Low density lipoprotein Receptor Related Protein 1	Protein forming a receptor found in the plasma membrane of cells involved in receptor-mediated endocytosis	Neurodegenerative diseases like Alzheimer’s disease, atherosclerosis, cancer, and CHD	15

Table S3 (continued)

#	Gene	protein	key functions	mutant phenotype	REF
33	Lrp2	Low density lipoprotein-related protein 2	Multiligand binding receptorfound in the plasma membrane of many absorptive epithelial cells and functions to mediate endocytosis of ligands leading to degradation in lysosomes or transcytosis	Donnai-Barrow syndrome, Dent Disease 1, and CHD	16
34	Ltbp1	Latent-transforming growth factor beta-binding protein 1	Belongs to the family of latent TGF-beta binding proteins (LTBPs) and the secretion and activation of TGF-betas is regulated by their association with latency-associated proteins and with latent TGF-beta binding proteins.	Geleophysic Dysplasia, Acromicric Dysplasia, and CHD	17
35	MEGF8	Multiple EGF Like Domains	The protein encoded by this gene is a single-pass type I membrane protein of unknown function that contains several EGF-like domains, Kelch repeats, and PSI domains	Carpenter syndrome 2 and CHD	18
36	Mmp21	Matrix Metallopeptidase 21	Involved in the breakdown of extracellular matrix for both normal physiological processes, such as embryonic development, reproduction, and tissue remodeling, and disease processes, such as asthma and tumor metastasis	Heterotaxy, Visceral, 7, Autosomal, Visceral Heterotaxy, and CHD	19
37	Myh10	Myosin Heavy Chain 10	Encodes non-muscle myosin II B (NM IIB), and is involved in tumor cell migration, invasion, extracellular matrix (ECM) production, and epithelial-mesenchymal transition (EMT)	May-Hegglin anomaly, developmental defects in brain, and CHD	20
38	Ndst1	Bifunctional heparan sulfate N-deacetylase/N-sulfotransferase 1	The encoded enzyme is a type II transmembrane protein that resides in the Golgi apparatus. The encoded protein catalyzes the transfer of sulfate from 3'-phosphoadenosine 5'-phosphosulfate to nitrogen of glucosamine in heparan sulfate	Mental Retardation, muscular hypotonia, epilepsy, postnatal growth deficiency, and CHD	21
39	Nek8	never in mitosis A-related kinase 8	a member of the serine/thereonine-specific protein kinase family related to NIMA (never in mitosis, gene A) of Aspergillus nidulans. The encoded protein may play a role in cell cycle progression from G2 to M phase	Congenital heart disease and nephronophthisis	22
40	PCSK5	Proprotein convertase subtilisin/kexin type 5	proprotein convertases that process latent precursor proteins into their biologically active products. This encoded protein mediates posttranslational endoproteolytic processing for several integrin alpha subunits	Congenital heart disease and Currarino syndrome	23

Table S3 (continued)

#	Gene	protein	key functions	mutant phenotype	REF
41	PDE2A	Phosphodiesterase 2A	dual-specificity for the second messengers cAMP and cGMP, which are key regulators of many important physiological processes. Plays an important role in growth and invasion of malignant melanoma cells	Congenital heart disease, Paroxysmal Dystonia and Chorea, Benign Hereditary	4
42	PKD1L1	Polycystin 1 Like 1, Transient Receptor Potential Channel Interacting	This gene encodes a member of the polycystin protein family containing 11 transmembrane domains, a receptor for egg jelly (REJ) domain, and a polycystin-1, lipoxygenase, alpha-toxin (PLAT) domain. The encoded protein may play a role in the male reproductive system.	Congenital heart disease	4
43	Pkdl	Polycystin-1	encodes a new member of the polycystin protein family designated polycystin-L.	Kidney disease, congenital heart disease	24
44	PLXND1	Plexin-D1	Plays an important role in cell-cell signaling, regulating the migration of a wide spectrum of cell types—including thymocytes in the medulla and endothelial cells—as well as ensuring the specificity of synapse formation. Required for normal development of the heart and vasculature	Moebius Syndrome and Conotruncal Heart Malformations. Congenital heart disease	2s5
45	Prdm1	PR domain zinc finger protein 1	The increased expression of the Blimp-1 protein in B lymphocytes, T lymphocytes, NK cell and other immune system cells leads to an immune response through proliferation and differentiation of antibody secreting plasma cells.	Congenital heart disease	26
46	Prickle 1	**Prickle planar cell polarity protein 1**	Encodes a nuclear receptor that may be a negative regulator of the Wnt/beta-catenin signaling pathway. The encoded protein localizes to the nuclear membrane and has been implicated in the nuclear trafficking of the transcription repressors REST/NRSF and REST4.	Congenital heart disease, progressive myoclonus epilepsy	27
47	Pskh1	**Serine/threonine-protein kinase H1**	May be a SFC-associated serine kinase (splicing factor compartment-associated serine kinase) with a role in intranuclear SR protein (non-snRNP splicing factors containing a serine/arginine-rich domain) trafficking and pre-mRNA processing.	Congenital heart disease	28

Table S3 (continued)

#	Gene	protein	key functions	mutant phenotype	REF
48	Ptk7	**Tyrosine-protein kinase-like 7**	Transduce extracellular signals across the cell membrane. The protein encoded by this gene is an intracellular domain with tyrosine kinase homology and may function as a cell adhesion molecule. This gene is thought to be expressed in colon carcinomas but not in normal colon, and therefore may be a marker for or may be involved in tumor progression. Four transcript variants encoding four different isoforms have been found for this gene.	Congenital heart disease	29
49	Robo1	Roundabout homolog 1	The protein encoded by ROBO1 is structurally similar to a Drosophila integral membrane protein which is encoded by the Drosophila roundabout gene (a member of the immunoglobulin gene superfamily) and is both an axon guidance receptor and a cell adhesion receptor, known to be involved in the decision by axons to cross the central nervous system midline	Dyslexia, congenital heart disease	30
50	Smad6	SMAD family member 6,	Cell signaling	Congenital heart disease, craniosynostos is	31
51	Smarca4	s ATP-dependent chromatin remodeler	Catalyzes helicase and ATPase activities and is thought to regulate transcription of certain genes by altering nearby chromatin structure. Part of the large ATP-dependent chromatin remodeling complex SWI/SNF, which is required for transcriptional activation of genes normally repressed by chromatin. Binds BRCA1, as well as regulate the expression of the tumorigenic protein CD44.[6]	Cancer, congenital heart disease	32

Table S3 (continued)

#	Gene	protein	key functions	mutant phenotype	REF
52	Snx17	Sorting nexin-17	Encodes a member of the sorting nexin family. Members of this family contain a phox (PX) domain, which is a phosphoinositide binding domain, and are involved in intracellular trafficking.	Congenital heart disease	33
53	Sufu	Suppressor of fused homolog	Encodes a component of the sonic hedgehog (SHH) / patched (PTCH) signaling pathway. Mutations in genes encoding components of this pathway are deleterious for normal development and are associated with cancer-predisposing syndromes	Congenital heart disease	34
54	Tab1	TGF-Beta Activated Kinase 1	Mediates various intracellular signaling pathways	Congenital heart disease	4
55	Tbc1d32	TBC1 Domain Family Member 32	Involved in sonic hedgehog signaling; interacts with and stabilizes cell cycle-related kinase. Alternative splicing results in multiple transcript variants	Congenital heart disease	35
56	TMEM67	Meckelin	The protein encoded by this gene localizes to the primary cilium and to the plasma membrane. The gene functions in centriole migration to the apical membrane and formation of the primary cilium. Multiple transcript variants encoding different isoforms have been found for this gene.	Congenital heart disease, Meckel syndrome type 3 (MKS3),nephronophthisis and Joubert syndrome type 6	36
57	Zbtb14	Zinc Finger and BTB Domain Containing 14	DNA-binding transcription factor activity *and* transcription regulatory region DNA binding.	Congenital heart disease, Holoprosencephaly 4 and Hermansky-Pudlak Syndrome.	4

Table S4. Proximity to telomeres vs. A+T content in genes causing congenital heart disease

#	gene ID	chr	gene locus near at	telomere locus near at	gene to telomere (Mbp); <50cM**?	A+T %	A+T >59%?	FL (bp)	rationale [REF]
1	Daw1	2	227Mb	242Mb	242-227=15	100-42=58	58	1683	[4]
2	DRC1	2	26 Mb	0 Mb	26-0 = 26	100-51=49	49	2461	[4]
3	Anks6	2	227Mb	242Mb	242-227=15	100-42=58	58	1683	[4]
4	Cfc1	2	130Mb	242Mb	242-130=112	100-56=44	44	1672	[4]
5	Lrp2	2	169 Mb	240 Mb	240-169=71	100-46=54	54	15657	[4]
6	Cxcr4	2	136 Mb	240 Mb	240-136=104	100-46=54	54	1668	[4]
7	Cml5/nat8	2	73	0	73	100-53=47	47	1085	[4]
8	Ltbp1	2	33 Mb	0 Mb	33-0=33	100-46=54	54	5294	[4]
9	Snx17	2	27 Mb	0 Mb	27-0=27	100-56=44	44	2357	[4]
10	CCDC39	3	180 Mb	198 Mb	198-180=18	100-36=64	64	3841	[4]
11	PLXND1	3	129 Mb	198 Mb	198-129=69	100-63=37	37	6913	[4]
12	Robo1	3	79 Mb	0 Mb	79-0=79	100-45=55	55	6927	[4]
13	Cc2d2a	4	15 Mb	0 Mb	15-0=15	100-46=54	54	1682	[4]
14	ADAMTS6	5	65 Mb	180 Mb	180-65=115	100-43=57	57	7309	[4]
15	DNAH5	5	13 Mb	0 Mb	13-0=13	100-44=56	56	15781	[4]

Table S4. Continued

	gene ID	chr	gene locus near at	telomere locus near at	gene to telomere (Mbp); <50cM**?	A+T %	A+T >59%?	FL (bp)	rationale [REF]
16	Foxjl	5	170Mb	181Mb	181-170=11	100-57=43	43	2321	4
17	Jbts17	5	37 Mb	0 Mb	37-0=37	100-39=61	61	11140	4
18	Lox	5	122 Mb	181 Mb	181-122=59	100-40=60	60	5076	4
19	Ndst1	5	150 Mb	181 Mb	181-150=31	100-57=43	43	8011	4
20	Tbc1d32	6	121 Mb	171 Mb	171-121=50	100-37=63	63	5101	4
21	Prdm1	6	106 Mb	170 Mb	170-106=64	100-46=54	54	5148	4
22	Ptk7	6	43 Mb	0 Mb	43-0=43	100-60=40	40	4222	4
23	PKD1L1	7	47 Mb	0 Mb	47-0=47	100-51=49	49	9105	4
24	DNAH11	7	21 Mb	0 Mb	21-0=21	100-45=55	55	14343	4
25	TMEM67	8	Q22.2	Q24.3	51	30, 34	64	4678	4
26	DnaI1	9	34 Mb	0 Mb	34 – 0 = 34	100-52=48	48	2529	4
27	PCSK5	9	76 Mb	138Mb	138-76=62	100-47=53	53	5788	4
28	Ift74	9	26 Mb	0 Mb	26-0=26	100-35=65	65	5325	4
29	ARMC4	10	27 Mb	0 Mb	27-0= 27	100-43=57	57	3603	4
30	Mmp21	10	125 Mb	133 Mb	133-125=8	100-54=46	46	1919	4
31	Sufu	10	102 Mb	131 Mb	131-102=29	100-57=43	43	5016	4
32	PDE2A	11	72 Mb	134 Mb	134 – 72 = 52	100-48=52	52	3765	4
33	Dync2h1	11	103Mb	135Mb	135-103=32	100-38=62	62	13683	4
34	CEP290	12	88 Mb	132 Mb	132-88=44	100-36=64	64	7824	4
35	Lrp1	12	57 Mb	133 Mb	133-57=76	100-60=40	40	14923	4
36	Prickle 1	12	42 Mb	132 Mb	132-42=90	100-46=54	54	5878	4
37	Frem2	13	38 Mb	113 Mb	113-38=75	100-45=55	55	16122	4
38	Pkdl	13	100 Mb	133Mb	133-100=33	100-53=47	47	2790	4
39	Hectd1	14	31 Mb	107 Mb	107-31=76	100-44=56	56	9445	4
40	Kif7	15	89 Mb	101 Mb	101-89=12	100-64=36	36	4567	4
41	Acan	15	88 Mb	101 Mb	101-88=13	100-57=43	43	8549	4
42	Smad6	15	66 Mb	101 Mb	101-66=35	100-59=41	41	3828	4

Table S4. Continued

#	gene ID	chr	gene locus near at	telomere locus near at	gene to telomere (Mbp); <50cM**?	A+T %	A+T >59%?	FL (bp)	rationale [REF]
43	Dyx1c1	15	55 Mb	101 Mb	101-55=46	100-40=60	60	1993	4
44	Ift140	16	1.5Mb	0 Mb	1.5-0=1.5	100-60=40	40	5232	4
45	Pskh1	16	68 Mb	90 Mb	90-68=22	100-60=40	40	3497	4
46	Cep110	16	19 Mb	0 Mb	19-0=19	100-38=62	62	5418	4
47	Dctn5	16	23 Mb	0 Mb	23-0= 23	100-45=55	55	10954	4
48	Nek8	17	28 Mb	83 Mb	83-28=55	100-56=44	44	3573	4
49	Myh10	17	8 Mb	0 Mb	8-0=8	100-49=51	51	7614	4
50	Ap2b1	17	35 Mb	83 Mb	83-35=48	100-46=54	54	5658	4
51	Zbtb14	18	5 Mb	0 Mb	5-0=5	100-40=60	60	3475	4
52	MEGF8	19	42 Mb	58 Mb	58-42=16	100-64=36	36	10936	4
53	Fuz	19	49 Mb	58 Mb	58-49=9	100-62=38	38	1728	4
54	Dnm2	19	10 Mb	0 Mb	10-0=10	100-61=39	39	3621	4
55	Smarca4	19	10 Mb	0 Mb	10-0=10	100-61=39	39	5577	4
56	Ap1b1	22	29Mb	50Mb	50-29=21	100-59=41	41	4165	4
57	Tab1	22	39 Mb	50 Mb	50-39=11	100-60=40	40	3198	4

Table S5. Genes associated with syndromic and non-syndromic thoracic aortic aneurysm

#	gene ID	chr	gene locus near at	telomere locus near at	gene to telomere (Mbp); <50cM**?	A+T %	A+T >59%?	FL (bp)	rationale [REF]
1	Acta2	10	89 Mb	133 Mb	133-89=44	100-52=48	48	1349	37
2	Bgn	X	153 Mb	156 Mb	156-153=3	100-70=30	30	2353	37
3	Col1a2	7	94 Mb	159 Mb	159-94=65	100-55=45	45	5072	37
4	Col3a1	2	189 Mb	242 Mb	242-189=53	100-52=48	48	5490	37
5	Col5a1	9	134 Mb	137 Mb	137-134=3	100-60=40	40	8442	37
6	Col5a2	2	189 Mb	242 Mb	242-189=53	100-48=52	52	6829	37
7	Efemp2	11	65 Mb	135 Mb	135-65=70	100-62=38	38	1920	37
8	Eln	7	74 Mb	159 Mb	159-74=84	100-61=39	39	3397	37
9	EMILIN1	2	27 Mb	0 Mb	27-0=27	100-68=32	32	3890	37
10	FBN1	15	48 Mb	102 Mb	102-48=54	100-47=53	53	11609	37
11	FLNA	X	154 Mb	156 Mb	156-154=2	100-62=38	38	8483	37
12	FOXE3	1	47 Mb	0 Mb	47-0=47	100-69=31	31	1768	37
13	LOX	5	122 Mb	181 Mb	181-122=59	100-40=60	60	5076	37
14	MAT2A	2	85 Mb	0 Mb	85-0=85	100-46=54	54	2817	37
15	MFAP5	12	8 Mb	0 Mb	8-0=8	100-40=60	60	2872	37
16	MYH11	16	15 Mb	0 Mb	15-0=15	100-55=45	45	6880	37
17	MYLK	3	123 Mb	198 Mb	198-123=75	100-49=51	51	10113	37
18	Notch1	9	136 Mb	138 Mb	138-136=2	100-63=37	37	9568	37
19	Prkg1	10	51 Mb	133 Mb	133-51=82	100-37=63	63	6615	37
20	SKI	1	2 Mb	0 Mb	2-0=2	100-61=39	39	6083	37
21	SLC2A10	20	46 Mb	64 Mb	64-46=18	100-50=50	50	4227	37
22	Smad2	18	47 Mb	80 Mb	80-47=33	100-40=60	60	34626	37
23	Smad3	15	67 Mb	102 Mb	102-67=35	100-51=49	49	6464	37
24	Smad4	18	51 Mb	80 Mb	80-51=29	100-41=59	59	8772	37
25	Tgfb3	14	76 Mb	107 Mb	107-76=31	100-52=48	48	3431	37
26	Tgfbr1	9	99 Mb	138 Mb	138-99=39	100-39=61	61	6492	37
27	Tgfbr2	3	30 Mb	0 Mb	30-0=30	100-47=53	53	4530	37

Table S6. Mouse CHD genes and associated human diseases

#	gene ID	chr	gene locus near at	telomere locus near at	gene to telomere (Mbp); <50cM**?	A+T %	A+T >59%?	FL (bp)	rationale [REF]
1	Armc4	10	27 Mb	0 Mb	27-0=27	100-43=57	57	3603	[4]
2	Ccdc39	3	180 Mb	198 Mb	198-180=18	100-36=64	64	3841	[4]
3	Dnaaf3	19	55 Mb	59 Mb	59-55=4	100-62=38	38	2242	[4]
4	Dnahl1	7	21 Mb	0 Mb	21-0=21	100-45=55	55	14343	[4]
5	Dnah5	5	13 Mb	0 Mb	13-0=13	100-44=56	56	15781	[4]
6	Dnail	9	34 Mb	0 Mb	34 – 0 = 34	100-52=48	48	2529	[4]
7	Drc1	2	26 Mb	0 Mb	26-0 = 26	100-51=49	49	2461	[4]
8	Dyx1c1	15	55 Mb	101 Mb	101-55=46	100-40=60	60	1993	[4]
9	Cc2d2a	4	15 Mb	0 Mb	15-0=15	100-46=54	54	1682	[4]
10	Cep290	12	88 Mb	132 Mb	132-88=44	100-36=64	64	7824	[4]
11	Jbts17	5	37 Mb	0 Mb	37-0=37	100-39=61	61	11140	[4]
12	Kif7	15	89 Mb	101 Mb	101-89=12	100-64=36	36	4567	[4]
13	Tmem67	8	93 Mb	144 Mb	144-93=51	100-37=63	63	4678	[4]
14	Bicc1	2	26 Mb	0 Mb	26-0 = 26	100-51=49	49	2461	[4]
15	Pkd1	13	100 Mb	133Mb	133-100=33	100-53=47	47	2790	[4]
16	Pkd1l1	7	47 Mb	0 Mb	47-0=47	100-51=49	49	9105	[4]
17	Dync2h1	11	103Mb	135Mb	135-103=32	100-38=62	62	13683	[4]
18	Ift140	16	1.5Mb	0 Mb	1.5-0=1.5	100-60=40	40	5232	[4]

References for supplementary materials

1. Chang YP, Liu X, Kim JD, Ikeda MA, Layton MR, Weder AB, Cooper RS, Kardia SL, Rao DC, Hunt SC, Luke A, Boerwinkle E, Chakravarti A. Multiple genes for essential-hypertension susceptibility on chromosome 1q. *Am J Hum Genet*. 2007;80:253-264
2. Padmanabhan S, Caulfield M, Dominiczak AF. Genetic and molecular aspects of hypertension. *Circ Res*. 2015;116:937-959
3. Rim JH, Choi YJ, Gee HY. Genomic landscape and mutational spectrum of adamts family genes in mendelian disorders based on gene evidence review for variant interpretation. *Biomolecules*. 2020;10
4. Li Y, Klena NT, Gabriel GC, Liu X, Kim AJ, Lemke K, Chen Y, Chatterjee B, Devine W, Damerla RR, Chang C, Yagi H, San Agustin JT, Thahir M, Anderton S, Lawhead C, Vescovi A, Pratt H, Morgan J, Haynes L, Smith CL, Eppig JT, Reinholdt L, Francis R, Leatherbury L, Ganapathiraju MK, Tobita K, Pazour GJ, Lo CW. Global genetic analysis in mice unveils central role for cilia in congenital heart disease. *Nature*. 2015;521:520-524
5. Taipale M, Kaminen N, Nopola-Hemmi J, Haltia T, Myllyluoma B, Lyytinen H, Muller K, Kaaranen M, Lindsberg PJ, Hannula-Jouppi K, Kere J. A candidate gene for developmental dyslexia encodes a nuclear tetratricopeptide repeat domain protein dynamically regulated in brain. *Proc Natl Acad Sci U S A*. 2003;100:11553-11558
6. Yu X, Ng CP, Habacher H, Roy S. Foxj1 transcription factors are master regulators of the motile ciliogenic program. *Nat Genet*. 2008;40:1445-1453
7. Smyth I, Du X, Taylor MS, Justice MJ, Beutler B, Jackson IJ. The extracellular matrix gene frem1 is essential for the normal adhesion of the embryonic epidermis. *Proc Natl Acad Sci U S A*. 2004;101:13560-13565
8. Dai D, Zhu H, Wlodarczyk B, Zhang L, Li L, Li AG, Finnell RH, Roop DR, Chen J. Fuz controls the morphogenesis and differentiation of hair follicles through the formation of primary cilia. *J Invest Dermatol*. 2011;131:302-310
9. D'Alonzo D, Emch FH, Shen X, Bruder E, De Geyter C, Zhang H. Hectd1 is essential for embryogenesis in mice. *Gene Expr Patterns*. 2019;34:119064
10. Wang X, Sha YW, Wang WT, Cui YQ, Chen J, Yan W, Hou XT, Mei LB, Yu CC, Wang J. Novel ift140 variants cause spermatogenic dysfunction in humans. *Mol Genet Genomic Med*. 2019;7:e920
11. Kubo T, Brown JM, Bellve K, Craige B, Craft JM, Fogarty K, Lechtreck KF, Witman GB. Together, the ift81 and ift74 n-termini form the main module for intraflagellar transport of tubulin. *J Cell Sci*. 2016;129:2106-2119
12. Hong H, Joo K, Park SM, Seo J, Kim MH, Shin E, Cheong HI, Lee JH, Kim J. Extraciliary roles of the ciliopathy protein jbts17 in mitosis and neurogenesis. *Ann Neurol*. 2019;86:99-115
13. Barakeh D, Faqeih E, Anazi S, M SA-D, Softah A, Albadr F, Hassan H, Alazami AM, Alkuraya FS. The many faces of kif7. *Hum Genome Var*. 2015;2:15006
14. Kattoor AJ, Goel A, Mehta JL. Lox-1: Regulation, signaling and its role in atherosclerosis. *Antioxidants (Basel)*. 2019;8
15. Shinohara M, Tachibana M, Kanekiyo T, Bu G. Role of lrp1 in the pathogenesis of alzheimer's disease: Evidence from clinical and preclinical studies. *J Lipid Res*. 2017;58:1267-1281
16. Collery RF, Link BA. Precise short sequence insertion in zebrafish using a crispr/cas9 approach to generate a constitutively soluble lrp2 protein. *Front Cell Dev Biol*. 2019;7:167
17. Dietzel E, Weiskirchen S, Floehr J, Horiguchi M, Todorovic V, Rifkin DB, Jahnen-Dechent W, Weiskirchen R. Latent tgf-beta binding protein-1 deficiency decreases female fertility. *Biochem Biophys Res Commun*. 2017;482:1387-1392

18. Lloyd DL, Toegel M, Fulga TA, Wilkie AOM. The drosophila homologue of megf8 is essential for early development. *Sci Rep*. 2018;8:8790
19. Guimier A, Gabriel GC, Bajolle F, Tsang M, Liu H, Noll A, Schwartz M, El Malti R, Smith LD, Klena NT, Jimenez G, Miller NA, Oufadem M, Moreau de Bellaing A, Yagi H, Saunders CJ, Baker CN, Di Filippo S, Peterson KA, Thiffault I, Bole-Feysot C, Cooley LD, Farrow EG, Masson C, Schoen P, Deleuze JF, Nitschke P, Lyonnet S, de Pontual L, Murray SA, Bonnet D, Kingsmore SF, Amiel J, Bouvagnet P, Lo CW, Gordon CT. Mmp21 is mutated in human heterotaxy and is required for normal left-right asymmetry in vertebrates. *Nat Genet*. 2015;47:1260-1263
20. Wang Y, Yang Q, Cheng Y, Gao M, Kuang L, Wang C. Myosin heavy chain 10 (myh10) gene silencing reduces cell migration and invasion in the glioma cell lines u251, t98g, and shg44 by inhibiting the wnt/beta-catenin pathway. *Med Sci Monit*. 2018;24:9110-9119
21. Reuter MS, Musante L, Hu H, Diederich S, Sticht H, Ekici AB, Uebe S, Wienker TF, Bartsch O, Zechner U, Oppitz C, Keleman K, Jamra RA, Najmabadi H, Schweiger S, Reis A, Kahrizi K. Ndst1 missense mutations in autosomal recessive intellectual disability. *Am J Med Genet A*. 2014;164A:2753-2763
22. Rajagopalan R, Grochowski CM, Gilbert MA, Falsey AM, Coleman K, Romero R, Loomes KM, Piccoli DA, Devoto M, Spinner NB. Compound heterozygous mutations in nek8 in siblings with end-stage renal disease with hepatic and cardiac anomalies. *Am J Med Genet A*. 2016;170:750-753
23. Mbikay M, Seidah NG, Chretien M, Simpson EM. Chromosomal assignment of the genes for proprotein convertases pc4, pc5, and pace 4 in mouse and human. *Genomics*. 1995;26:123-129
24. Vogel P, Read R, Hansen GM, Freay LC, Zambrowicz BP, Sands AT. Situs inversus in dpcd/poll-/-, nme7-/- , and pkd1l1-/- mice. *Vet Pathol*. 2010;47:120-131
25. Gitler AD, Lu MM, Epstein JA. Plexind1 and semaphorin signaling are required in endothelial cells for cardiovascular development. *Dev Cell*. 2004;7:107-116
26. Turner CA, Jr., Mack DH, Davis MM. Pillars article: Blimp-1, a novel zinc finger-containing protein that can drive the maturation of b lymphocytes into immunoglobulin-secreting cells. 1994. *J Immunol*. 2010;185:5-14
27. Katoh M, Katoh M. Identification and characterization of human prickle1 and prickle2 genes as well as mouse prickle1 and prickle2 genes homologous to drosophila tissue polarity gene prickle. *Int J Mol Med*. 2003;11:249-256
28. Brede G, Solheim J, Prydz H. Pskh1, a novel splice factor compartment-associated serine kinase. *Nucleic Acids Res*. 2002;30:5301-5309
29. Mossie K, Jallal B, Alves F, Sures I, Plowman GD, Ullrich A. Colon carcinoma kinase-4 defines a new subclass of the receptor tyrosine kinase family. *Oncogene*. 1995;11:2179-2184
30. Massinen S, Wang J, Laivuori K, Bieder A, Tapia Paez I, Jiao H, Kere J. Genomic sequencing of a dyslexia susceptibility haplotype encompassing robo1. *J Neurodev Disord*. 2016;8:4
31. Timberlake AT, Choi J, Zaidi S, Lu Q, Nelson-Williams C, Brooks ED, Bilguvar K, Tikhonova I, Mane S, Yang JF, Sawh-Martinez R, Persing S, Zellner EG, Loring E, Chuang C, Galm A, Hashim PW, Steinbacher DM, DiLuna ML, Duncan CC, Pelphrey KA, Zhao H, Persing JA, Lifton RP. Two locus inheritance of non-syndromic midline craniosynostosis via rare smad6 and common bmp2 alleles. *Elife*. 2016;5
32. Dsouza NR, Zimmermann MT, Geddes GC. A case of coffin-siris syndrome with severe congenital heart disease and a novel smarca4 variant. *Cold Spring Harb Mol Case Stud*. 2019;5
33. Stockinger W, Sailler B, Strasser V, Recheis B, Fasching D, Kahr L, Schneider WJ, Nimpf J. The px-domain protein snx17 interacts with members of the ldl receptor family and modulates endocytosis of the ldl receptor. *EMBO J*. 2002;21:4259-4267

34. Merchant M, Vajdos FF, Ultsch M, Maun HR, Wendt U, Cannon J, Desmarais W, Lazarus RA, de Vos AM, de Sauvage FJ. Suppressor of fused regulates gli activity through a dual binding mechanism. *Mol Cell Biol*. 2004;24:8627-8641
35. Adly N, Alhashem A, Ammari A, Alkuraya FS. Ciliary genes tbc1d32/c6orf170 and sclt1 are mutated in patients with ofd type ix. *Hum Mutat*. 2014;35:36-40
36. Morgan NV, Gissen P, Sharif SM, Baumber L, Sutherland J, Kelly DA, Aminu K, Bennett CP, Woods CG, Mueller RF, Trembath RC, Maher ER, Johnson CA. A novel locus for meckel-gruber syndrome, mks3, maps to chromosome 8q24. *Hum Genet*. 2002;111:456-461
37. Brownstein AJ, Ziganshin BA, Kuivaniemi H, Body SC, Bale AE, Elefteriades JA. Genes associated with thoracic aortic aneurysm and dissection: An update and clinical implications. *Aorta (Stamford)*. 2017;5:11-20
